# Is Aging a Disease? A Critical Review Within the Framework of Ageism

**DOI:** 10.7759/cureus.54834

**Published:** 2024-02-24

**Authors:** Víctor Manuel Mendoza-Núñez, Ana Belén Mendoza-Soto

**Affiliations:** 1 Unidad de Investigación en Gerontología, Universidad Nacional Autónoma de México, Mexico, MEX; 2 Posdoc Research of Biology, Centro Interdisciplinario de Investigación para el Desarrollo Integral Regional - Sinaloa, Mexico, MEX

**Keywords:** ageing & disease, stereotypes, discrimination, hallmarks, ageism

## Abstract

Ageism is a type of discrimination characterized by negative social representations of old age and aging, with prejudices and stereotypes that cause rejection and marginalization of older adults, generally considering them as fragile and unproductive. For this reason, it is recognized as one of the main enemies of healthy aging, especially when it arises from the scientific and professional fields. In this sense, the proposals promoted by some researchers regarding the World Health Organization (WHO) classifying aging as a disease goes against the healthy aging approach. In this sense, we consider that there is no theoretical or scientific support to classify aging as a disease, so we must advocate before the WHO so that aging is eliminated within its disease classification codes. In this framework, this review proposes the concept of "hallmarks of ageism” defined as the characteristics, representations and attitudes of rejection and discrimination towards aging, old age and older people, at the political and institutional, scientific or professional, technological and digital, social, family and personal levels, which are presented in an articulated and structured manner. For this reason, it is essential to comprehensively identify and analyze the “hallmarks of ageism”, in order to propose programs that include strategies and public policies that promote “anti-ageism” as a counterproposal to the "hallmarks of aging", whose biological changes related to aging are intended to be comparable to chronic non-communicable diseases.

## Introduction and background

In recent years, a biologist and medicalized approach to aging has been promoted. In this sense, López-Otín et al. (2013), published a review on "the hallmarks of aging", proposing nine mechanisms as the biochemical and molecular processes that cause aging: (i) genomic instability, (ii) telomere attrition, (iii) epigenetic alterations, (iv) loss of proteostasis, (v) deregulated nutrient sensing, (vi) mitochondrial dysfunction, (vii) cellular senescence, (viii) stem cell exhaustion, and (ix) altered intercellular communication [1]. Likewise, geroscience defined as "an interdisciplinary field that aims to understand the relationship between aging and age-related diseases and disabilities" has reinforced this approach with the proposal of the so-called "main pillars of geroscience research", adding the inflammaging and oxidative stress to the hallmarks of aging, with the purpose of finding a treatment that modulates or counteracts said alterations to prevent chronic non-communicable diseases (CNCDs), such as diabetes, cardiovascular disease; chronic obstructive pulmonary disease; chronic kidney disease, cancer, osteoporosis, arthritis, Alzheimer’s disease, among others [2]. In this sense, it has even been pointed out that aging should be recognized as a disease by the United States Food and Drug Administration, in order to be able to treat it and avoid or delay the appearance of CNCDs [3]. For this reason, some researchers proposed to the World Health Organization (WHO) that aging and old age be recognized and included as a disease in the International Classification of Diseases 11th Revision (ICD-11). The proposal was approved in 2019, to enter into force in 2022 [4-6]; however, said approval generated broad rejection in the geriatric and gerontological field, because it was included in the term "Old age" under general symptoms (code MG2A), the WHO responded to this claim by replacing the term “Old age” with “Ageing associated decline in intrinsic capacity” [7-9]. However, although the proposal is qualified with said change, the codes XT9T (Ageing-related) and MG2A (Ageing-associated decline in intrinsic capacity) are maintained in the recently published ICD-11 [10], for which reason the WHO currently considers aging as a disease. However, this proposal has been questioned, pointing out that aging is not a disease and, as such, should not be treated; rather, is a process of losses and gains, where we seek to minimize the losses that occur over time, and optimize the gains at the individual level to achieve a better functional healthspan. In this sense, it is important to note that the WHO recognizes functional capacity as a key element of healthy aging, defined as “the process of developing and maintaining the functional ability that enables well-being in older age” [11].

In this context, the purpose of this review is to present an analysis of the concept of human aging, justifying why it should not be considered as a disease, since this approach could be considered as the hallmark of ageism.

## Review

Methodology

This narrative review included published articles that defined aging as a biopsychosocial process, as well as those that proposed it as a disease. The critical analysis was framed in the concept of ageism. PubMed and Google Scholar databases were used to compile the most relevant articles using keywords such as “definition of aging,” “aging is a disease,” “types of ageism”. Only relevant articles that met the requirements that defined the aging process, assumed aging as a disease, as well as the different types of ageism that linked the concept of aging with the disease were included. The initial selection process identified 552 articles, and after applying the inclusion criteria, 73 were considered relevant for the review.

Human aging

There is no agreed definition of human aging, in this regard, biological aging is the one that has been most attempted to define, supported by aging theories, of which it has been reported that there are more than 300 [12]. Nevertheless, Troen (2003) points out five characteristics common to aging in mammals: (i) exponential growth in mortality is observed with advancing age after the reproductive stage; (ii) changes in the composition of the tissues, with increasing age, there is a decrease in muscle and bone mass, an increase in adipose tissue, the development of lipofuscin deposits and crosslinking with structural proteins such as collagen, due to processes that occur in aging such as oxidation and glycosylation, and an increase in AGEs (advanced glycation end products); (iii) progressive decrease in the physiological capacity of all systems; (iv) decreased adaptive response to environmental stimuli, so that aging occurs with a gradual decrease in the ability to maintain homeostasis; and (v) increased vulnerability to disease, as age-related changes impair cell function, leading to tissue and organ dysfunction, ultimately triggering systemic disease [13].

Likewise, Lemoine (2020) in a review and analysis of the definition of biological aging has pointed out five characteristics commonly used in biogerontology to define aging: (i) structural damage, (ii) functional decline, (iii) depletion of a reserve required to compensate for the decline, (iv) typical phenotypic changes or their cause, and (v) increasing probability of death (or disease). In this regard, he clarifies that any definition of aging must include some of these characteristics. In this sense, the author carried out an exhaustive review and analysis of the main definitions of biological aging considering these criteria. As a result of this review, he points out that “aging should be considered to be a binary process, with a progressively shifting balance between degradation (promotive) and compensation (protective) mechanisms” [14].

On the other hand, Goluveb (2021) specifies the difference between the definition of real (essential) and nominal aging, highlighting that the latter focuses on observable characteristics. Therefore, the research is oriented towards the description, analysis and explanation of the pathways and mechanisms that link aging with its characteristics and manifestations, without considering the real definition [11]. Likewise, Cohen et al. (2020), demonstrated the lack of consensus on the definition of biological aging at an international symposium on the biology of aging, in which 37 researchers responded to a survey on the basics of biological aging, regarding when aging begins, if aging is biologically programmed, the biological mechanisms of aging, whether aging is or can be quantifiable, and whether aging is treatable. The survey revealed a significant disagreement on fundamental issues of the conceptual framework of aging, for which the following was concluded: (i) if there is no agreement on definitions and mechanisms of aging, how can we identify, measure or know it?, what is it?, what are we measuring?; (ii) how to evaluate possible anti-aging interventions? if there is no precision in the concept of aging; (iii) is it feasible to extrapolate the biological aging processes of other species to humans considering their complexity? The paradox of classifying aging as a disease and wanting to provide treatment to prevent it is how is it possible to diagnose and cure a process inherent to life [15,16].

In this context, we can point out that there is no consensus in the gerontological field regarding the age of onset of human aging, some authors focus on time relative to age, pointing out that aging begins from birth and even from conception. Assuming that "I get older every day", this approach is very limited, since the process of human aging is much more complex. In this sense, Leonard Hayflick establishes that “it is not the simple passage of time; but the manifestation of biological events that occur over a period of time, which characterizes aging”, hence he points out that “aging occurs over time, but not by the passage of time”. For this reason, Hayflick defines biological aging as "a stochastic process that occurs systemically after reproductive maturity in animals that reach a fixed size in adulthood and is caused by the increasing loss of molecular fidelity that ultimately exceeds the capacity for repair", thus increasing vulnerability to pathological or age-associated diseases [17-19].

Due to the aforementioned, we consider that it is not pertinent to extrapolate the conceptual framework of molecular, cellular, tissue, apparatus and systems aging, to the complexity of human aging, since from the human life cycle approach, it is not logical to assume that the growth and development of humans runs parallel to biological aging. In this sense, most of the cells in our body did not exist five or ten years ago, even two days ago, hence the cells renew themselves over time, especially during childhood and adulthood, so cells of our body in recent years are younger, the paradoxical question would be: if most of our cells change over time and are therefore younger, then do we rejuvenate over time? Of course, the answer is no, since aging at the molecular and cellular level is not necessarily the equivalent of human aging. For this reason, we can assert that children grow and develop before reaching adulthood, but do not age until after adulthood. Therefore, our research group has defined human aging as a "gradual and adaptive process, characterized by a relative decrease in the reserve and biological response to the demands to maintain or recover homeostasis, due to morphological, physiological, biochemical, psychological and social, caused by the genetic load and accumulated wear and tear in the face of the challenges that the person faces throughout his history in a certain environment, which manifests itself after maturity with individualized physical, psychological and social changes, increasing vulnerability to infectious and CNCDs” [20]. In this regard, it is necessary to clarify that the study of aging should not be limited to the medical-biological field, but rather must have a multidisciplinary and multidimensional approach, considering psychological, social, geographical and cultural aspects. In this sense, psychosocial gains are also observed during aging, such as (i) greater knowledge and experience; (ii) greater capacity for free time and leisure; (iii) in some cases savings and better economic situation; (iv) a high percentage of politicians and businessmen are older adults (v); language is preserved, (v) procedural memory and priming tend to be well-preserved, and only episodic long-term memory and working memory typically decline [21-23]. The approach to active aging and the empowerment of older adults, supported by activity theory, has been decisive in increasing health span in old age, in addition to recognizing the economic and social contribution of older adults [24,25].

On the other hand, with pragmatic purposes of clinical and community intervention, we assume that aging begins from the fifth decade of life, around the age of 45, considering that biological, bodily, psychological, and social changes are evident after that stage of life, characterized by a gradual physiological and functional decline in the majority of the population, increasing the risk of CNCDs [20]. This does not mean that we are unaware that human aging is a complex and multifactorial process, and therefore individualized, nor of the importance of physical, psychological and social health care at other stages of life (childhood, adulthood) that corresponds to other specialists.

Some researchers in the field of aging biology have promoted and advocated for the recognition of aging as a disease. In this sense, Bulterijs et al. (2015) point out that the medical definition of disease is any abnormality of a bodily structure or function, other than those that arise directly from a physical injury and the disorder has a specific cause and recognizable signs and symptoms, and therefore, there is no denying the fact that aging is a "damaging abnormality of bodily structure and function", not only does aging lend itself to being characterized as a disease, but the advantage of doing so is that, by rejecting the apparent fatalism of the "natural" label, better legitimizes medicine's efforts to eliminate it or get rid of the undesirable conditions associated with it. The authors also note that aging being recognized as a disease would encourage grant-making agencies to increase funding for aging research and to develop biomedical procedures to slow the aging process [4].

Calimport and Bentley (2019) highlighted the importance of the WHO classifying aging as a disease, supporting the "anti-aging" approach [5]. Likewise, Khaltourina et al. (2020) emphasized the achievement of the WHO recognizing aging and old age as diseases [6], however, the claim of several geriatricians and gerontologists has been pointed out, which led to its exclusion at the same time in the ICD-11 as a disease [7-9]. However, it is very disconcerting and unheard of that the WHO maintains aging as a disease in its classification [10], which can be classified as a hallmark of institutional ageism.

Regarding the above, we totally disagree, since aging is a process inherent to living beings. In this sense, aging can only be stopped by completely eliminating metabolism or the macromolecules that store information, that is, by eliminating what makes life alive. As Golubev (2021) has pointed out, aging is not a nullity or a disease, but a trait selected in evolution to lead living beings to an opportune death. Therefore, it has emerged concomitantly with the emergence of life and has been since then an indispensable condition for biological evolution to face [14].

On the other hand, the clinical and sociosanitary implications of recognizing or classifying aging as a disease must also be considered: (i) At what age should aging be diagnosed as a disease? from birth? 45 years?, at 65 years?, and when should one clinically detect any of the alterations of the so-called "hallmarks of aging"?; (ii) how to classify old people with healthy lifestyles, "do they not age" and therefore "would always be young"?; (iii) what would happen to life insurance and medical expenses, since some of the "hallmarks of aging" could be detected at some point?; (iv) would there be “healthy and sick old people” or would only the sick “be old” and the healthy “be young?; (v) classifying aging as a disease increases ageism in all areas, personal, family, scientific, social and political.

Ageism and disease

Butler (1969) proposed the concept of ageism as a type of discrimination, characterized by stereotypes, prejudices, stigmatization and rejection of old age and the old [26]. This approach and line of research has been widely accepted throughout the world, recognizing its implications in the fields of health, social inclusion, well-being, quality of life and human development during aging and old age.

In this regard, the WHO specifies that ageism is made up of three dimensions: (i) stereotypes (thoughts), (ii) prejudice (feelings) and (iii) discrimination (actions or behavior). Stereotypes and prejudices can be positive or negative, and discrimination can be implicit or explicit [27]. Ageism can be institutional and/or political, scientific, social, family and personal. In this sense, Marques et al. (2020), in a systematic review of the determinants of ageism, identified the intrapersonal, interpersonal/intergroup and institutional levels as the main determinants [28]. On the other hand, Kang and Kim (2022) in a systematic review of the relationship between ageism and psychological well-being found a negative association between ageism and older adults' psychological well-being [29].

The studies by Levy et al. regarding the beneficial effect of positive self-perception of old age on longevity, functional health, and recovery from disability are widely known [30-32]. In this sense, in a systematic review carried out by them, in which 422 studies from 45 countries around the world were analyzed, which included over 7 million participants, the authors found that ageism led to significantly worse health outcomes in 95.5% of the studies and 74.0% of the 1,159 ageism-health associations examined [33].

Likewise, Levy et al. (2020) found that the one-year cost of ageism was $63 billion for older from the United States during one year [34]. Also, our research group found a positive influence of the self-perception of old age on the effect of a healthy aging program [35].

Hallmarks of ageism

**Figure 1:**
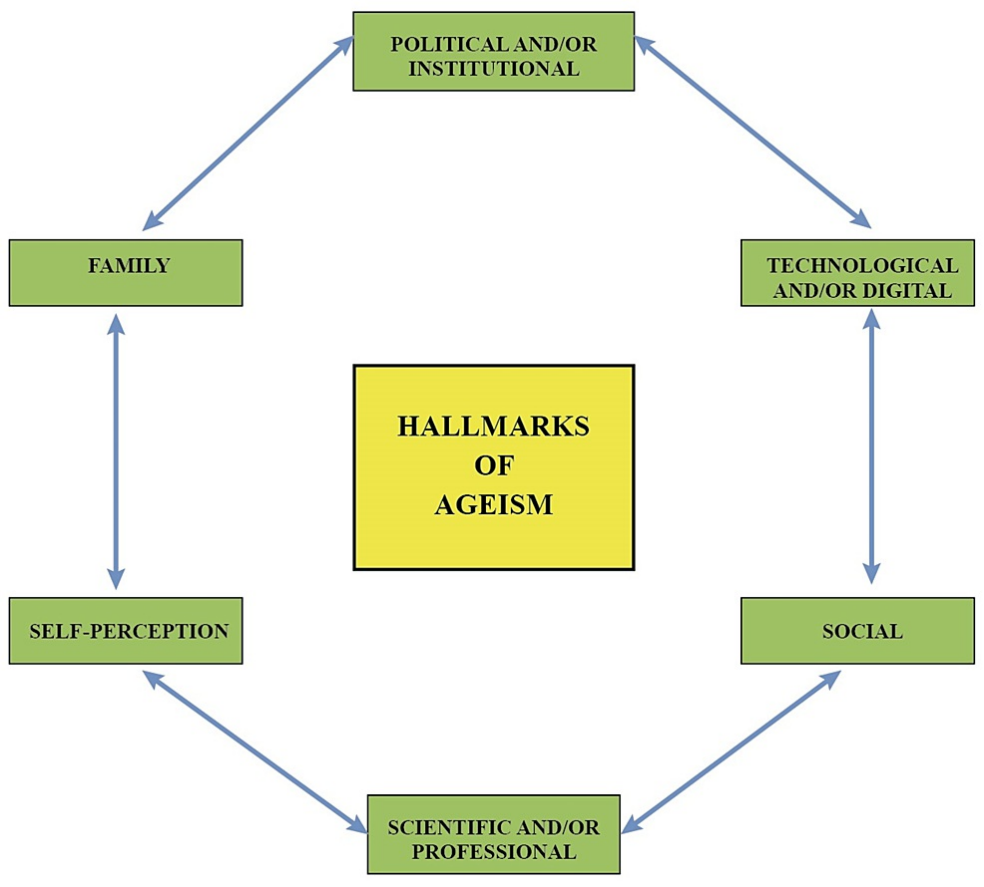
Hallmarks of ageism Political and/or institutional: (i) Aging is a disease, (ii) Population aging is “gray tsunami” or “silver tsunami, (iii) Population aging is an economic burden, (iv) Aging as political utilitarianism; Scientific and/or profesional: (i) anti-aging medicine, (ii) Emphasis on the diagnosis and treatment of geriatric syndromes and physical and cognitive impairment, (iii) Geroscience promotes that aging is considered a disease; Technological and/or digital: (i) A high percentage of older adults do not have access or knowledge for the use of computers, internet and smartphones; (ii) A high percentage of older adults are excluded from the use of virtual social networks (WhatsApp, Facebook  Instagram and TikTok), (iii) Telegerontology is not used to promote healthy aging; Social: (i) Stereotypes of aging are promoted by the media, (ii) Overvaluation of youth and rejection of the image of old age, (iii) Invisibility of the contributions of the elderly in the economy, (iv) Lack of recognition of the social capital represented by the population of older adults, (v) lack of opportunities for human development during aging; Family: (i) Lack of care or physical and/or psychological abuse, (ii) Overprotection or unnecessary care, (iii) Usurpation of the autonomy, (iv) Exclusion in the use of technology and virtual social networks; Self-perception: Self-ageism is the result of the other hallmarks of ageism (Mendoza-Nuñez VM & Mendoza-Soto AB, 2022).

We propose the concept of “hallmarks of ageism” as a response to the implications and negative repercussions of the biologist approach of classifying aging as a disease. The hallmarks of ageism refer to the characteristics, representations and attitudes of rejection and discrimination towards aging, old age and the old, at the political and institutional, scientific or professional, technological and digital, social, family and personal levels (Figure 1).

Political and/or Institutional

In the political and institutional sphere, the following can be highlighted as examples: (i) the classification of aging as a disease by the WHO [10]; (ii) some international organizations classify population aging as "gray tsunami" or "silver tsunami", as a catastrophic warning [36,37], without considering the potential of older adults for their own development and their current social contribution; (iii) pointing out the aging of the population as an economic burden [38]; (iv) political utilitarianism of some governments, who propose undifferentiated benefactor programs for electoral purposes, without considering the human and social capital of the older people for their own development; (v) differentiated and discriminatory care of health services towards adults older, in some cases it is rejection, lack of interest or empathy or with a negative approach to deterioration during health care [39], this was more evident during the COVID-19 pandemic, where a discriminatory quarantine for the elderly was proposed as the only alternative, promoting isolation, depression and in some cases rejection of older adults by other family members [40,41], in some countries the priority of installing respirators in the case of complications was questioned, considering older adults as non-priority.

Scientific and/or Professional

In the scientific field, the most obvious examples that promote ageism can be highlighted: (i) the so-called "anti-aging medicine", with thousands of followers in the world, who investigate and promote the use and consumption of supposed anti-aging treatments [42]. Likewise, many geriatricians focus mainly on the diagnosis and treatment of geriatric syndromes with an emphasis on frailty, with a vision of old age as deterioration [43]. Also, as has been pointed out, Geroscience has promoted the approach of classifying aging as a disease, in order to receive drug treatment [2].

Technological and/or Digital

A type of ageism that has emerged in recent decades is technological and digital, since a high percentage of older adults do not have access and/or knowledge of the use of current technology, which is much more accentuated in developing countries [44]. In this regard, the Economic Commission for Latin America and the Caribbean (CEPAL, 2019) reported that less than 25% of older adults (≥60 years) use the Internet, with very varied frequencies, Uruguay 24.5%, Chile 22.6%, Peru 9.2%, Ecuador 8.8%, Mexico 8.0%, Paraguay 7.7%, El Salvador 6.6%, and Honduras 6.1% [45]. Likewise, the use of virtual social networks is focused on certain age groups, for example, "TikTok" is a platform used mainly by young people and adults, however, it has been pointed out that it could be a good alternative to promote healthy aging and counteract ageism among groups of older adults, in addition to being inclusive with the current media [46]. On the other hand, telegerontology is an alternative to remote health care, for which the availability and use of technology are essential. In this regard, telegerontology has focused mainly on the support of caregivers and family members for the control of patients with Alzheimer's disease [47,48], however, telegerontology has great development potential for healthy aging.

Social

In the social sphere, the following can be pointed out: (i) the prejudices and stereotypes of old age and discrimination represented in the media (including virtual social networks) [49], which intensified during the COVID-19 pandemic [50]; (ii) overvaluation of youth and rejection of the image of old age. The most obvious example is the so-called "anti-aging medicine" that proposes to cure aging to achieve eternal youth [51]; (iii) the invisibility of the contributions of the elderly in the economy. In this regard, Fernandez-Ballesteros et al. (2011) in a study carried out on a sample of the non-dependent Spanish population of 55 to 75 years, showed that said population is contributing to society with its productive unpaid activities of about 106 billion Euros (adult and child caregiving; shopping, purchasing; personal/household administrative management and messages; household work; handwork; and formal volunteering) [52]; (iv) lack of recognition of the human and social capital that the elderly population represents to achieve and maintain healthy aging [53,54]; (v) lack of opportunities for human development during aging and old age [55,56].

Family

Ageism in the family environment is much more frequent than what is recognized by the elderly and the members of the family, above all because it is mostly of a cultural, implicit and structured type, since it is a consequence of the social representations of the aging and old age, the media, public policies, culture and traditions, so it can be generally accepted by the elderly and normalized by the family and society. In this sense, it can be expressed in a negative way with rejection, lack of care and even physical abuse and consider them as a burden. Likewise, it can be of a positive type through overprotectionism (unjustified physical and psychological care) that can anticipate physical limitations and usurpation of autonomy, assuming that because they are older adults they have deterioration, cannot be independent and therefore cannot take appropriate decisions [57,58]. As has been pointed out, in recent decades a type of family exclusion of older adults related to knowledge and use of technology has emerged, since young family members assume that technology, such as the use of smartphones, apps, computers, Internet and virtual social networks (WhatsApp, Facebook, Instagram and TikTok), is for young people and not for older adults.

Self-Perception

Self-ageism is a consequence of all the other hallmarks of ageism, since public policies, institutions, social representations, the media, the family and the approaches to the study of aging are determinant factors of the self-perception of aging and old age, in addition to sex, education, socioeconomic level, physical and mental condition, functionality, occupation, social support networks, limitations in functionality, ethnic group, marital status and living alone or with family, and the use of technology and virtual social networks [28,59].

How to combat ageism

The World Health Organization (WHO, 2021) has reported the following data on population aging in the world: “(i) between 2015 and 2050, the proportion of the world's population over 60 years will nearly double from 12% to 22% ; (ii) by 2020, the number of people aged 60 years and older will outnumber children younger than 5 years; (iii) in 2050, 80% of older people will be living in low- and middle-income countries; (iv) the pace of population aging is much faster than in the past, and (v) all countries face major challenges to ensure that their health and social systems are ready to make the most of this demographic shift”. In this regard, the challenge implied by the higher prevalence and incidence of CNCDs is highlighted [60]. Likewise, the WHO (2015) in the World Report on Aging and Health specifies the concept of healthy aging as "the process of developing and maintaining the functional ability that enables wellbeing in older age", highlighting that healthy aging is different to the state of health, since the key element of healthy aging is the functional capacity, characterized by the attributes related to health that allow a person to BE AND DO WHAT IS IMPORTANT for them, so healthy aging is contextual and the environment and support networks are determinants [61]. Subsequently, the Decade of Healthy Aging (2021-2030) was proposed (WHO, 2020), in which four areas of action were established, so that healthy aging is jointly strengthened: (i) “change how we think, feel and act towards age and aging”, with the purpose of counteracting ageism and its negative impact on health and well-being; (ii) “ensure that communities foster the abilities of older people”, to enhance their abilities through a community gerontology approach; (iii) “deliver person-centred integrated care and primary health services that are responsive to older people”, with the aim of optimizing community resources, considering individualized care, “between peer” social support networks, self-care and self-management; (iv) “provide access to long-term care for older people who need it”, to improve the quality of care, wellbeing and quality of life of older adults with functional limitations [62].

Therefore, the proposal to classify aging as a disease is outside the world's conceptual and reference framework, which is paradoxical, since on the one hand, the WHO recognizes the social capital represented by older people and promotes an approach to enhance physical, psychological and social capacities by recognizing intrinsic capacity, and on the other hand, it has yielded to pressure from some research groups to classify aging as a disease [10], which we have classified as one of the main hallmarks of ageism.

Regarding interventions to reduce ageism, in a systematic review and meta-analysis carried out by Burnes et al. (2019), in which they analyzed 63 studies, observed that the best effect in reducing ageism was observed when combined programs with educational and intergenerational contact components were included [63].

On the other hand, the WHO (2021) published the Global Report on Ageism, and defines it as “the stereotypes (how we think), prejudice (how we feel) and discrimination (how we act) directed towards people on the basis of their age”. It can be institutional, interpersonal or self-directed. Institutional ageism refers to the laws, rules, social norms, policies and practices of institutions that unfairly restrict opportunities and systematically disadvantage individuals because of their age. Interpersonal ageism arises in interactions between two or more individuals, while self-directed ageism occurs when ageism is internalized and turned against oneself [27]. In this sense, it points out that one in two older adults is subject to some type of ageism, with repercussions on people's health, well-being and human rights. Likewise, older people who are rejected or discriminated against present a shorter lifespan, worse physical and mental health, slower recovery from disability, and cognitive decline. It also negatively affects their quality of life, increases their social isolation and loneliness, increases poverty, restricts their ability to express their sexuality, and may increase the risk of violence and abuse against older people [27].

In this context, we propose that the fight against ageism must be comprehensive, considering as components: (i) human rights; (ii) education; (iii) recognizing the human and social capital of older adults as a demographic bonus; (iv) permanent “anti-ageism” campaign in the media; (v) empowerment of older adults; (vi) person-centered care; and (vii) implementation of the Integrated Care for Older People (‎ICOPE).

Human Rights

The United Nations (1982) at the World Assembly on Aging proposed to all Governments to incorporate the Principles for Older Persons: Independence, Participation, Care, Self-fulfilment and Dignity, which were formally incorporated in 1991 [64], however, many of the rights have only remained on paper and have not been implemented. Likewise, the Organization of American States (OEA, 2015) proposed the "Inter-American Convention on the Protection of the Human Rights of Older Persons", which entered into force in 2017 [65], however up to now no have been made significant changes. For this reason, we must advocate that human rights be the transversal element to eradicate ageism.

Education

Education in all environments, in home (with the family), community and social, is the key element to change the negative view of aging and old age and counteract ageism. In this regard, in a systematic review of interventions to reduce ageism against older adults, in which they analyzed the results of 63 studies with a total sample of 6,124 participants, it was reported that combined interventions with education and intergenerational contact showed the largest effects on change of negative attitudes towards older adults [63]. On the other, hand, it is necessary to include issues on aging and the social capital that older adults represent in the study plans and programs of all levels, especially in professional training programs for medical, nursing, social work, psychology, law, pedagogy, communication sciences, architecture, anthropology, gerontology, among others [58,66].

Social Capital of the Older People (Gerontological Bonus)

Gerontological human and social capital is the set of real and potential resources that older adults have, they are linked to the knowledge and experience that each person possesses, the networks of social relationships they have and the recognition they obtain from others. It is made up of knowledge, skills, experience, and attitudes, such as leadership, solidarity, reciprocity, and empathy, coupled with peer-to-peer social support networks [67]. In this sense, in a systematic review carried out by Coll-Planas et al. (2017), that social capital interventions had a favorable impact on mental and physical health, mortality and use of health-related resources [68]. For this reason, we propose that the population group of functional, independent and autonomous older adults should be considered as a "gerontological demographic bonus", recognizing the human and social capital that they represent for their own development and the direct and indirect economic and social contribution that they continue to do.

Permanent Anti-ageism Campaign in the Media

One of the strategies that are currently being promoted to counteract ageism is the establishment of “anti-ageism” campaigns. For this purpose, the WHO created a platform within the framework of the decade of healthy aging, which includes a space to promote a permanent campaign to combat ageism [69]. Another highly relevant initiative is the LeadingAge® association platform, where you can find a wide variety of scientific and outreach documents, as well as experiences on “anti-ageism” intervention, a document "Anti-Ageism Quick Guide: Changing the Conversation” [70].

Empowerment

Empowerment is a participatory development process through which individuals, communities and organizations achieve greater control over their lives and environment, acquiring rights and new goals in their lives, coupled with a reduction in social marginalization, involves self-empowerment, to control, to their own power, to self-confidence, to their own decision, to a dignified life according to one's values, to the ability to fight for one's rights, to independence, to own decision-making [71].

The main strategies at the community level to achieve empowerment are training, leadership development, support for the establishment of participatory assistance policies and programs, education, organization of associations, cooperatives, creation of empowerment collectives, such as "Peers for Progress Global Network” [72,73].

In order to exercise empowerment efficiently and effectively, it is essential that older adults are included in decision-making, to ensure that the use of public and private resources for gerontological programs responds to the real needs of the population. Likewise, participatory group work should be promoted, including older adults in community intervention programs that are developed in their locality [74].

Person-Centered Care

American Geriatrics Society defined “Person-centered care” (P-CC) as “individuals’ values and preferences are elicited and, once expressed, guide all aspects of their health care, supporting their realistic health and life goals. P-CC is achieved through a dynamic relationship among individuals, others who are important to them, and all relevant providers. This collaboration informs decision-making to the extent that the individual desires” [75]. The P-CC recognizes that older people are more than the set of their disorders or diseases; it takes into account their particular experiences, needs and preferences. It also addresses the person's health and social care needs rather than focusing on isolated disorders or symptoms. This approach also takes into account the context of the person's daily life, including the impact of their health and their needs on those close to them and on the community. In this regard, the P-CC recognizes the priorities and capacities of the elderly, to recover and maintain functionality.

Implementation of Integrated Care for Older People (ICOPE)

ICOPE is a strategy proposed by the WHO (2019), in which the guidelines are specified to detect priority conditions associated with decreased intrinsic capacity and the environment, to promote healthy aging in the community, with a care approach focused on the person (the person's health and social care needs) and not on isolated illnesses or symptoms [76]. In this regard, our research group has developed a model of "gerontological nuclei" for peer-to-peer community support for older adults, recognizing the relevance of the human and social capital that older adults represent as a key element of support networks social for healthy aging [77]. ICOPE promotes the participation and empowerment of older adults, families and communities. In this sense, it includes training for older adults, families, volunteers and caregivers, so that they actively participate in community programs that allow the identification of the main conditions associated with the decrease in intrinsic capacity to maintain, prolong or recover functional capacity: (i) meet basic needs; (ii) maintain mobility; (iii) establish and maintain social relationships; (iv) learn, grow and make decisions; and (v) contribute to society [78,79]. Therefore, the WHO implicitly establishes that stopping the curve of deterioration or decline of intrinsic capacity and benefits and optimizing functional capacity can be considered the strategy to combat ageism and not classify aging as a disease.

## Conclusions

Ageism is one of the main enemies of healthy aging, especially when it arises from the scientific and professional fields. In this regard, the proposal to classify aging as a disease is opposed to the healthy aging approach. In this sense, there is no theoretical or scientific support to classify aging as a disease, so we must advocate before the WHO that aging be eliminated within its classification codes. On the other hand, we propose the concept of the "hallmarks of ageism" as a counterproposal of the "hallmarks of aging" with which it tries to justify that the biological, biochemical and molecular changes related to aging correspond to those of disease. However, the researchers who defend this approach recognize that healthy lifestyles prevent or delay the occurrence of said alterations (hallmarks of aging), so that aging per se, does not promote said alterations, but rather are the unhealthy lifestyles are risk factors for such alterations.

We define the hallmarks of ageism as the characteristics, representations and attitudes of rejection and discrimination towards aging, old age and the old, at the political and institutional, scientific or professional, technological and digital, social, family and personal levels, which are presented in an articulated and structured manner. For this reason, it is essential to comprehensively measure the hallmarks of ageism, in order to propose global programs that include all the mechanisms that promote “anti-ageism”. In this regard, it is suggested that these programs be implemented at a pilot level in "anti-ageism" communities, so that they are an example and have a multiplier effect, as occurs with the global age-friendly cities program proposed by the WHO.
